# Analysis of the Meiotic Segregation in Intergeneric Hybrids of Tilapias

**DOI:** 10.1155/2012/817562

**Published:** 2012-06-18

**Authors:** Etienne Bezault, Xavier Rognon, Frederic Clota, Karim Gharbi, Jean-Francois Baroiller, Bernard Chevassus

**Affiliations:** ^1^UMR110 Cirad-Ifremer INTREPID, 34398 Montpellier, France; ^2^INRA, UMR1313 Génétique Animale et Biologie Intégrative, 78352 Jouy-en-Josas, France; ^3^Department of Biology, Reed College, Portland, OR 97202, USA; ^4^AgroParisTech, UMR1313 Génétique Animale et Biologie Intégrative, 75231 Paris, France; ^5^Institute of Evolutionary Biology, School of Biological Sciences, University of Edinburgh, Edinburgh EH9 3JT, UK

## Abstract

Tilapia species exhibit a large ecological diversity and an important propensity to interspecific hybridisation. This has been shown in the wild and used in aquaculture. However, despite its important evolutionary implications, few studies have focused on the analysis of hybrid genomes and their meiotic segregation. Intergeneric hybrids between *Oreochromis niloticus* and *Sarotherodon melanotheron*, two species highly differentiated genetically, ecologically, and behaviourally, were produced experimentally. The meiotic segregation of these hybrids was analysed in reciprocal second generation hybrid (F2) and backcross families and compared to the meiosis of both parental species, using a panel of 30 microsatellite markers. Hybrid meioses showed segregation in accordance to Mendelian expectations, independent from sex and the direction of crosses. In addition, we observed a conservation of linkage associations between markers, which suggests a relatively similar genome structure between the two parental species and the apparent lack of postzygotic incompatibility, despite their important divergence. These results provide genomics insights into the relative ease of hybridisation within cichlid species when prezygotic barriers are disrupted. Overall our results support the hypothesis that hybridisation may have played an important role in the evolution and diversification of cichlids.

## 1. Introduction

Interspecific hybridisation has been suggested to be an important evolutionary force that generates biological diversity by the recombination of genetic material among divergent lineages [1–3]. Hybridisation has been shown to facilitate adaptation, the emergence of evolutionary novelty, and the isolation of new species [4–7]. The role of introgressive hybridisation has also been shown in case of human-mediated evolution and especially domestication [8]. For instance, interspecific hybridisation has been widely used for aquaculture purposes in a large variety of species [9]. 

Convincing evidence suggests that inter-specific hybridisation has played an important role during evolution and diversification of cichlid fish [10]. Some cichlid adaptive radiations may have been initiated through hybridisation between distantly related lineages, forming a “hybrid swarm,” such as the radiations of Lakes Victoria [11], Malawi [12], and Makgadikgadi [13]. Hybridisation can also occur later during the process of radiation between divergent or already diverged species, forming “syngameon”, as suggested for some Lake Tanganyika lineages [14–16]. The vast majority of the studies conducted so far on cichlid speciation and the potential influence of hybridisation have focused on the East African Great Lakes radiations, especially the Haplochromines. However, the Tilapiines *sensu lato* [17, 18] appear to be an extremely interesting group to study the evolutionary role of hybridisation. Several cases of introgressive hybridisation have been recorded in the wild, under natural conditions, either during the process of adaptive radiation, as in Cameroonian crater lake [19], or during paleoenvironmental fluctuations [20]. Several cases have also been reported following anthropogenic perturbations, either between sympatric species after important habitat modifications such as dam building [21] or between allopatric species after introduction of species outside their natural distribution area [22, 23]. Furthermore, numerous inter-specific hybrids have been produced for aquaculture [24], mainly among *Oreochromis* species [9, 25, 26]. The production of tilapia hybrids has two main purposes: the production of monosex populations by crossing between species with opposite sex determination system [27–31] and the improvement of phenotypic traits, such as body colour, growth, or tolerance to environmental conditions [24]. However, it is only for the latter that introgressive hybridisation is performed, with the production of breeding population of hybrid origin, aiming at being propagated and selected throughout successive reproductive events. This has been conducted mostly crossing pairs of closely related species (e.g.,* Oreochromis niloticus* and *O. aureus*, [32]), but also using more complex crossing schemes involving multiple species (e.g., Red Florida stain, between *Oreochromis urolepis hornorum*, *O. mossambicus*, *O. niloticus,* and *O. aureus*) [33].

Theoretically, while the evolutionary potential of a hybrid lineage is ultimately dependent on its ability to successfully occupy a peak of the local adaptive landscape through the advantage conferred by its original combination of phenotypic traits, hybrid propagation initially requires the maintenance of a stable gene pool through generations. Most of theoretical and empirical studies are focussing on the adaptive role of hybridisation (see review by Stelkens and Seehausen [34]); however very few studies have investigated the transmission of hybrid genomes across generations.

Various hybrid meiotic mechanisms deviating from classical diploid Mendelian segregation and inheritance have been reported, especially in fish [35, 36]. Such mechanisms may be classified in four main categories: (1) hybridisation can induce variable ploidy levels by the production of nonreduced (diploid) gametes; this mechanism is likely to lead to the isolation of polyploidy lineages as, for example, in *Barbus* species [37]; (2) hybridisation can induce clonal gynogenetic reproduction by the suppression of syngamy (i.e., absence of fusion and elimination of male pronucleus) and the mitotic or meiotic restoration of diploidy, such as observed in *Poecilia formosa* [38]; (3) hybridogenesis can be achieved by selective meiosis, in which the genome of one of the parents (generally the paternal one) is preferentially eliminated from the gametes leading to the next generation, as described in hybrid females of *Poeciliopsis* [39]; (4) modification of recombination within the hybrid genomes, caused by the slight structural divergences between the associated genomes, might affect the homogeneity of the hybrid gene pool, leading, for example, to the maintenance of a mosaic hybrid genome and a fitness deficit [40–43].

To investigate hybrid meiotic segregation in cichlids, we previously produced an intergeneric hybrid between two highly divergent species using *in vitro* fertilisation: the Nile tilapia, *Oreochromis niloticus,* and the black-chinned tilapia, *Sarotherodon melanotheron *[44]. All hybrids were found to be viable and fertile for at least three generations [44]. The divergence between the two species is estimated between 6.4 Myrs and 21.4 Myrs using the age of radiation of *Oreochromis* and Oreochromines as an approximation [18]. Additionally, these two species show high levels of differentiation in morphology, ecology, behaviour, and physiology. *O. niloticus * is a maternal mouthbrooder, fast growing and freshwater stenotopic species, whereas *S. melanotheron* is a paternal mouthbrooder, slow growing, brackish water eurytopic species. Selecting two highly differentiated parental species for the experimental hybridisation potentially increases the possibility of generating original trait association in hybrids compared to the parental species (including transgressive characters) but also increases the likelihood for reproductive incompatibility or unusual meiotic mechanisms, compared to hybridisation between closely related or sister species. Thus, these inter-generic hybrids represent an original and well-adapted model to study the association and segregation of parental genes within cichlid hybrid genomes.

Microsatellite markers provide valuable tools for a wide range of genetic investigations, including species comparison using PCR primers developed in one species and cross-amplified in closely related taxa [45, 46]. Here we took advantage of the availability of a large number of markers cloned in *O. niloticus* [47] positioned onto the tilapia genetic maps [48–51] and their high rate of cross-species amplification among tilapias [52], to study the mechanism of hybrid meiotic segregation in experimental hybrids between *O. niloticus* and *S. melanotheron*. We tested (1) whether hybrid meiotic segregations follow diploid Mendelian inheritance and (2) whether this model of meiotic segregation allows the maintenance of a stable hybrid gene pool across generations.

## 2. Material and Methods

### 2.1. Biological Material

The meiosis of first generation hybrids (F1) between the 2 parental species *O. niloticus* (On) and *S. melanotheron* (Sm) was studied for both reciprocal hybrid crosses (♀ On × ♂ Sm called G1 and ♀ Sm × ♂ On called G′1) and both sexes. Analysis was conducted on backcross (BC) and second-generation hybrid (F2) families. While F2 crosses allow studying hybrid meiosis for both parents in each family, BC progeny allowing the study of only one hybrid meiosis per family can be more informative with respect to the parental origin of alleles, and further allow the analysis of a parental meiosis, which provide an internal pure-species control segregation.

Four backcross families performed on *O. niloticus* (due to its better known reproduction biology and greater ease to be manipulated) were analysed to study the meiotic segregation of both reciprocal hybrid types and both sexes (♀ G1, ♂ G1, ♀ G′1 & ♂ G′1), and the pure-species segregation of *O. niloticus* in both sexes (♀ On & ♂ On) (Table 1). In addition, two different hybrid F2 families (♀ G′1 × ♂ G′1 and ♀ G1 × ♂ G′1) were analysed to look at the segregations and allele associations within a full hybrid genome (Table 1). Finally, an independent pure *S. melanotheron* family (♀ Sm & ♂ Sm; P-Sm) was studied to provide information on the meiotic segregation of the other parental species (Table 1). For each family, 50 randomly sampled individuals were analysed.

### 2.2. Microsatellites Markers

Microsatellites markers were selected from published markers isolated in *O. niloticus* [47] and successfully amplified in both *O. niloticus* and *S. melanotheron* [52]. Markers were selected based on cross-amplification efficiency, polymorphism among and within *O. niloticus* and *S. melanotheron*, and their position on the genetic map of tilapias [48–51]. The analysis of the meiotic segregation was conducted using a total of 30 microsatellite markers distributed across the tilapia genome, allowing to compare the segregation of both punctual genomic locations, represented by single independent loci, as well as larger genomic segments represented by two to four linked loci. Overall, the 30 selected markers represented 15 of the 24 linkage groups (LGs) defined in *O. niloticus* genetic map, 8 of each were represented by more than one marker. Two unmapped loci were also included in the analysis (Table 2).

### 2.3. Genotyping of Microsatellites

Genomic DNA was extracted from fin clips stored in ethanol using phenol-chloroform protocol [53]. Genotypes were obtained by PCR amplification using radioactively (P^33^) labelled primers and 6% acrylamide gel electrophoresis [53, 54]. For each microsatellite marker, optimal amplification conditions, namely annealing temperature and MgCl_2_ concentration for coamplification of alleles from heterospecific origin, were obtained from a cross-species amplification study in over 15 species of African cichlids, including both parental target species [52]. Specific PCR conditions for each locus are indicated in Table 2.

All parents used to produce the experimental progeny were genotyped first at all selected loci to identify informative markers for each family. Each family was genotyped for all informative loci in the entire set of optimised markers (*n* = 30), whereas the F2 hybrid progeny was only genotyped across the restricted set of independent markers (*n* = 14) (Table 2; details in supplementary Table  S1 available online at doi:10.1155/2012/817562). 

### 2.4. Statistical Analysis

#### 2.4.1. Genetic Diversity

The test of amplification efficiency and the estimates of genetic diversity within and across the two-target parental species have been conducted over the set of individuals analysed during cross-species amplification study and the set of pure parents and grandparents of the seven experimental progenies included in the analysis of meiotic segregation [52]. For each locus, the number of observed alleles was recorded for each parental species, as well as the number of shared alleles between them. The presence of null alleles in the studied loci was estimated across all breeder individuals, based on the repetitive occurrence of a nonamplification result in parents, and/or the significant departure from Mendelian inheritance associated with a pattern characteristic of the segregation of at least one nonamplified allele (i.e., based on the overall pattern of alleles segregation in the entire progeny). For each species, the proportion of polymorphic loci (*P* < 0.95) and the mean allele number per loci were calculated. The number of shared alleles, between *O. niloticus* and *S. melanotheron*, as percent of total number of observed alleles, was calculated per locus and across loci. For the purpose of this study, the existence of shared alleles between parental species and/or their low allelic diversity can potentially lead to cases where marker segregation is not fully informative (e.g., when both parents are heterozygous for the same alleles). These cases were identified based on the genotyping of all parent individuals, prior to the progeny genotyping.

#### 2.4.2. Analysis of Meiotic Segregation

The analysis of the meiotic segregation was conducted for each individual parent and family, as well as across all pure and hybrid segregations, considering either each locus independently or collectively. In order to accurately account for false positives due to multiple testing, a sequential Bonferroni correction was applied [55], considering the multiple tests performed either within a given progeny/breeder-segregation across independent loci or for a given locus or pair of loci across the independent tested segregations/families.

Accordance of the observed segregations to Mendelian expectations was tested using a Chi^2^ test to detect possible distortion of segregation. Sequential Bonferroni correction of *P*-value for multiple tests was performed across loci separately within each cross. The balance of global meiotic transmission of both parental alleles was tested within each hybrid segregation using a Chi^2^ test across all loci within each breeder segregation. Homogeneity of reciprocal backcrosses was tested by comparison of genotypic distributions between families for each locus using Chi^2^ tests, with global tests combining these results across loci for each families comparison using Fisher's method [56]. Comparisons were carried out between progeny from the same hybrid type (G1 or G′1), or the same hybrid way (♀ or ♂), and overall cases. Pairwise linkage analysis was performed using LinkMFex [57] using an LOD score of 3 as threshold for significance. Linkage associations with a 2 ≤ LOD Score < 3 were considered as suggestive. Recombination rates were compared using a Chi^2^ test.

## 3. Results

### 3.1. Microsatellite Diversity

A high percentage of the markers were polymorphic in both species (Table 2), with a slightly lower diversity in *S. melanotheron* (77%) than in *O. niloticus* (97%). A similar pattern was observed for the mean allelic diversity per locus and species (2.8 and 4.3, resp.). Null alleles were detected for a total of 7 markers, with a higher frequency in *S. melanotheron* (*n* = 6) than *O. niloticus* (*n* = 1). Markers showed a low percentage of shared alleles (7.7%) between the two parental species involved in the hybridisation, which allowed the accurate identification of the genetic origin of the alleles segregating in hybrid meiosis. Overall, the level of polymorphism present in both parental species population was sufficient to obtain a high frequency of informative segregation across the different types of progeny: 55% of the pure *O. niloticus* segregations appeared informative, 43% of *S. melanotheron* and 79.5% of hybrids segregations (90% in BC and 57% in F2—details in Supplementary Table  S1). 

### 3.2. Analysis of Meiotic Segregation

A total of 4 out of 232 segregations (1.7%) showed significant evidence of segregation distortion (*P* < 0.05 after correction for multiple testing). Three significant tests were observed in hybrid genomes (2 in ♂ G′1, UNH-008 & UNH-216, and 1 in ♀ G1, UNH-197) and a single significant test in pure species (♀ *S. melanotheron*, UNH-135) (details in Supplementary Table  S1).

Out of the 8 hybrid parents analysed, equal transmission of alleles from both parental and species origin was observed in the vast majority of the cases (*n* = 7), representing at least one meiotic segregation of each hybrid cross (G1 and G′1) and sex (Table 3). The hypothesis of equal transmission of parental species alleles was rejected in a single hybrid male (BC-D family), which globally undertransmitted its paternal alleles of *O. niloticus* origin (Table 3). This was consistent with the results from the locus-level analysis for this individual, which revealed evidence of unequal allele transmission for 2 significant loci from 3 different LGs after Bonferroni correction, always in the direction of undertransmission of *O. niloticus* paternal alleles (details in Supplementary Table  S1).

When we considered allelic distributions observed among backcross progeny, according to hybrid type and/or sex, only the comparison between hybrid males exhibited significant heterogeneity (*P* < 0.01) (Table 4). However, the comparison between the 2 types of hybrids, G1 versus G**′**1, and globally among the 4 different sex and hybrid types did not reveal any evidence of deviation from homogeneity.

Genetic linkage was detected in 16 cases (Table 5), of which 14 cases were expected from previous publications [48–51], but 2 being unexpected among these studies. The large majority of expected linkage associations (73%) were confirmed, including the 2 unexpected cases (i.e., UNH-008 & UNH-146, and UNH-154 & UNH-207, with LOD > 3 in 5 and 7 segregations, resp.). Three expected associations were not observed: UNH-008 & UNH-103 (LG 17) showed no cosegregation, whereas UNH-102 & UNH-138 (LG 16) and UNH-130 & UNH-197 (LG 23) showed a suggestive linkage (LOD > 2), but only in one case for each of these pairs. One unexpected linkage (UNH-008 & UNH-124) was significant in 6 parents. The other unexpected association (UNH-106 & UNH-207) was only found in one parent out of 3. Finally one unmapped marker, UNH-117, was assigned to the LG 5 with a significant linkage in 2 parents. The occurrence of these linkage associations was checked comparatively between the 2 pure and the hybrid genomes. All but one was significant in *S. melanotheron* parents, and every linkage significantly established in *O. niloticus* showed significant linkage in hybrid parents.

Recombination rates were compared using a Chi^2^ test between parents of opposite sex and/or genetic types in pure and hybrid individuals (see details in Supplementary Table  S3). Out of 16 pairs of linked loci (i.e., for which a significant linkage has been detected in at least one parent), heterogeneity of recombination rate in at least one comparison was observed for 2 pairs of loci (i.e., UNH-125 & UNH-138 (LG16) and UNH-131 and UNH-135 (LG3)). These 2 cases where homogeneity of recombination rate was rejected were detected in comparisons involving pure-species parents. In both cases, evidence of heterogeneity was observed within species (in *O. niloticus* comparisons, between sexes and among males, resp.) as well as between species (i.e., between *O. niloticus* and *S. melanotheron*). No evidence of significant heterogeneity was observed among hybrid meioses.

## 4. Discussion

We have characterized meiotic segregation in experimental intergeneric hybrids between two highly differentiated Oreochromine cichlid species, *O. niloticus* and *S. melanotheron*, from which no natural hybrid has ever been reported, due to ecological and reproductive behaviour divergence, even if sympatric in some basins of West Africa [58–60]. Our analysis permitted to track the origin and transmission of alleles across 17 independently mapped anchors distributed over the tilapia genome, including 8 genomic segments represented by 2 to 4 linked loci to survey variation of recombination between hybrids, parental species, and/or sexes.

All hybrid parents showed systematic exclusion of the alleles from each of the two parental species (i.e., hybrid parents transmitted either the *O. niloticus* allele or the *S. melanotheron * allele but never both or none). The strict disjunction of both *O. niloticus* and *S. melanotheron* alleles at each locus confirms locus homology between these two species and indicates the diploid state of these hybrids, at both the sequence and genome organisation level. The balanced segregations of loci lead to Mendelian transmission of both parental species alleles to the next generation for both reciprocal ways of crosses (G1 & G′1). A stable hybrid gene pool was maintained through meiosis, although segregation distortions were observed in very few cases (1.7%). As we observed no preferential allele elimination, neither in terms of parental species nor cross-direction (maternal/paternal origin), the observed departures from expected Mendelian inheritance may be due to local meiosis irregularities (i.e., male G′1 hybrid from BC-D), rather than a general effect of hybridisation.

The vast majority of linkage associations between markers expected in *O. niloticus* based on existing genetic maps [48–51] were confirmed in this study. Moreover these associations appeared well conserved in *S. melanotheron* and hybrids. This is consistent with a high level of synteny conservation between tilapia species, as previously observed between the closely related *O. niloticus* and *O. aureus* [50] and further extends this observation to distantly related species among Oreochromines. Recombination rates appear very similar between sexes as well as among hybrids and pure species. We did not observe a contraction of the genetic map as may be expected in hybrids because of reduced recombination due to structural genomic differences between parental species [50]. Taken together our results allow us to reject the hypothesis of meiotic irregularities or specific meiotic processes (i.e., nonhomoploid biparental transmission) in the hybrid between *O. niloticus* and *S. melanotheron*.

Our study also allowed us to demonstrate Mendelian transmission during the F1 hybrid meiosis, independent of the sex and/or direction of crosses (G1 or G′1), leading to the maintenance of a stable and balanced hybrid gene pool through generations. These results, especially the strict disjunction of parental alleles at each locus and the conservation of linkage groups without genetic map contraction, suggest a close genomic structure between both parental species and their hybrids [50, 51]. The analysis of phenotypic traits segregating in hybrids is particularly interesting in this context, especially considering the numerous divergent traits between the two parental species, such as morphology, reproductive behaviour, and physiological adaptive capacity. Physiological traits, for example, growth and salinity tolerance, show intermediate phenotypes in F1 hybrids [44, 61]. In experimental conditions, the fertility of hybrids has been established up to the F2 and viability up to F3 (for both hybrid and backcross progeny—unpublished data). Fertility problems have been previously reported in interspecific fish hybrids (see review by Bartley et al. [9]), not only at the initial hybrid stage (i.e., sterility in F1 generally due to ploidy perturbations) but also in subsequent hybrid generations, as in the case of hybrid models where high fitness is observed in early hybrids (F1) followed by a strong fitness breakdown in subsequent generations (F2+). Such examples were observed in tilapia during a complex four-species crossing project between three *Oreochromis* and one *Sarotherodon* [49], where the viable and fertile two-way F1 hybrids led to four-way F2 hybrids unable to produce viable progeny [62]. Such rapid introgression of four genomes in only two generations might have exacerbated instability of the resulting gene pool leading to fertility problems. This phenomenon would have been avoided or strongly reduced in case of a two-way hybridisation and/or increasing the number of generations to mix the genomes, especially if it involved an introgressive (backcross) scheme and/or closely related parental species. In any case, the existence of a late fitness disruption in hybrids tends to suggest the absence of major genomic reorganisation between Oreochromines species, in favour of small genomic rearrangement(s), for example, microinversions and insertions, structural variations and/or divergent gene evolution. These elements can all be potentially responsible for the establishment of postzygotic isolation resulting from the increase of Dobzhansky-Muller genetic incompatibilities [63]. Over time, both premating and postmating incompatibilities are expected to accumulate between divergent lineages, with a relative rate depending on different parameters, including the geographical mode of speciation, the existence of sexual dimorphisms, and/or strong sexual selection, overall yielding to the “speciation clock” [64].

As cichlids are known to exhibit sexual selection, typically associated with male breeding colour polymorphism [65], theory predicts a faster loss of premating than postmating compatibility. In the species used in this study, pre-mating isolation is total, most notably due to the divergence of reproductive behaviour between the two parental species (i.e.,* O. niloticus* is a maternal mouthbrooder whereas *S. melanotheron* is a paternal mouth-brooder, which explains the need for *in vitro* fertilisation for the production of F1 hybrids between these species). Post-mating isolation also appears to be relatively weak and probably only due to drift in the absence of reinforcement mechanisms between the two parental species. This process might be different depending on the cichlid lineage considered, based on their rate of transition between reproductive systems. For instance, while Oreochromines exhibit maternal, paternal, and biparental mouth-brooding systems, the most species-rich lineage, Haplochromines, exhibits exclusively maternal mouth-brooding reproductive systems [66, 67]. The absence of pre-mating barrier based on reproductive behaviour among species would either be compensated by other component(s) of pre-mating isolation system, such as sexual selection mediated by male colour polymorphism, or represent an overall lower level of premating isolation, which would be prone to generate an increased rate of postzygotic accumulation (i.e., through reinforcement process).

Two recent studies have investigated the dynamics of hybridisation in Haplochromine cichlids [68]. While phenotypic novelty does increase with the genetic distance between parental hybridizing lineages [68], the accumulation of reproductive incompatibilities is also building up with divergence time [69]. This later study demonstrates that, along the axis of species divergence, pre-mating isolation is built up fast initially (i.e., due to mate choice and sexual selection) without increasing much later (e.g., due to the highly conserved courtship behaviour and the absence of change in reproductive system across the entire lineage), whereas post-mating incompatibilities only start accumulating at relatively later stages of divergence. Such a pattern may have led to “evolutionary viable” hybridisation between lineages diverging for a very long time (i.e., estimation of the hybrid unviability after 4.4–18.4 Myrs, depending on the molecular clock calibration used [69]). Considering the latest estimates of divergence time between the radiation of the entire Oreochromines tribe (12.8–21.4 Myrs) and the radiation of the genus *Oreochromis* (6.4–9.7 Myrs) [18], the experimental hybrids between *O. niloticus* and *S. melanotheron* stand at the later end of the continuum tested in Haplochromines. Our results then appear consistent with the findings drawn from hybridisation in Haplochromines, while adding an independent estimate from another major cichlid tribe. Additionally, the present study extends the previous results by demonstrating the maintenance of normal meiotic mechanisms in hybrids between highly divergent species, long after the completion of pre-mating isolation (i.e., once post-mating isolation would be expected to have already started accumulating).

The important role of interspecific hybridisation during the evolution of cichlids, especially the processes of adaptation and diversification, has been extensively discussed [10]. Hybridisation is thought to occur when populations are subjected to important environmental changes, such as anthropogenic perturbations [22, 23, 59, 70] and hydroclimatic changes [20], and when populations invade a new environment [10], enabling these populations to undergo rapid adaptive response and even radiation. Evidence suggests that many of the largest cichlid lake radiations may have been initiated and fuelled through hybridisation between distantly related lineages [11–13], which invaded the new empty environment and started interbreeding to form a hybrid swarm, predisposing the colonizing lineages to diversify. One of the crucial issues in the hypothesis of hybrid swarm origin of adaptive radiations is to identify the mechanism by which hybrid lineage(s) can be initiated and propagated. Demonstrating that hybridisation between highly distantly related Oreochromines species can lead to meiotic processes following diploid Mendelian segregation and the maintenance of a stable and recombining hybrid gene pool across generations appears to strongly support this hypothesis. Overall, this study provides functional insights into hybridisation in cichlids, when prezygotic barriers are disrupted, despite important divergence time between lineages, and therefore supports the idea that interspecific hybridisation has the potential to play an important role in the evolution and diversification of cichlids.

## Supplementary Material

The supplementary material provides the details about Informative marker segregations and segregation distortions observed in the different families studied (Table S1); the adjustment to Mendelian expectations of the genotype distributions (Table S2); and the comparisons of the recombination rate between the different genetic types (Table S3).

## Figures and Tables

**Table 1:** Description of the experimental families analysed, including the type of cross, the genetic origin of breeders (i.e., pure *O. niloticus*) (On) or *S. melanotheron* (Sm), and reciprocal 1st generation hybrid G1 (♀ On × ♂ Sm) and G′1 (♀ Sm × ♂ On) and the number of studied individuals.

Families	Type of crossing	Breeders		No. Ind.
		Female	Male	
BC-A	Backcross	Hybrid Gl	*O. niloticus *	50
BC-B	Backcross	*O. niloticus *	Hybrid Gl	50
BC-C	Backcross	Hybrid G′l	*O. niloticus *	50
BC-D	Backcross	*O. niloticus *	Hybrid G′l	50
F2-A	Hybrid F2	Hybrid G′l	Hybrid G′l	50
F2-B	Hybrid F2	Hybrid Gl	Hybrid G′l	50
P-Sm	Pure Cross	*S. melanotheron *	*S. melanotheron *	50

**Table 2:** Microsatellite loci analysed with indications of GenBank accession number, repeat structure, and linkage group according to *O. niloticus * [47, 48, 51], optimised PCR conditions established from cross-species amplification study of microsatellites across 15 African cichlid species (labeled primer “*”, annealing temperature and Magnesium concentration (mM)—Bezault et al., [52]); the size range and diversity of alleles within each parental species as well as the number of shared alleles between them and the presence of null allele (N) have been estimated from the set of individuals used for cross-priming analysis and the parents of all families studied here; the set of independent loci analysed in all backcross and F2 hybrid families are indicated in **bold**.

Loci	GenBank accession	Structure	Linkage group	PCR conditions	Range (bp)	Allelic diversity		Shared alleles
						*O. niloticus*	S. *melanotheron *	
**UNH-008**	G31346	perfect	17	R* 56/1.2	196–236	3	2	0
**UNH-102**	G12255	perfect	16	R* 50/1.2	132–185	4	4	0
UNH-103	G12256	perfect	17	R* 48/1.2	171–260	3	4	0
UNH-106	G12259	compound	3	R* 50/1.2	115–189	4	2 + N	1
UNH-115	G12268	compound	3	F* 50/1.5	100–146	3	1	0
UNH-117	G12270	interrupted		R* 54/1.2	108–146	1	2	1
UNH-123	G12276	perfect	12	F* 48/1.2	142–232	6	2	0
UNH-124	G12277	perfect	4	F* 54/1.2	295–324	4	1	0
UNH-125	G12278	compound	16	R* 48/1.5	134–198	6	4	2
**UNH-129**	G12282	interrupted	8	R* 48/1.2	180–253	7	4	1
UNH-130	G12283	perfect	23	R* 50/1.2	174–242	6	1 + N	0
**UNH-131**	G12284	perfect	3	F* 48/2.0	283–303	4	2 + N	0
UNH-132	G12285	perfect	9	R* 52/1.2	100–134	2	1 + N	0
UNH-135	G12287	interrupted	3	R* 50/1.5	124–284	6	4	1
UNH-138	G12290	perfect	16	R* 48/1.5	144–250	7	2	0
**UNH-142**	G12294	interrupted		F* 48/1.2	142–192	3	2	0
**UNH-146**	G12298	interrupted	4	F* 60/1.0	111–149	3	3	1
**UNH-149**	G12301	perfect	5	R* 48/1.5	143–225	4	3	0
**UNH-154**	G12306	perfect	6	R* 50/1.2	98–176	8	5	2
UNH-159	G12311	perfect	2	R* 55/1.2	205–267	5 + N	3	1
**UNH-162**	G12314	perfect	4	R* 48/1.5	125–252	6	2	0
UNH-169	G12321	interrupted	5	R* 54/1.2	124–240	8	4 + N	1
UNH-173	G12325	perfect	13	F* 55/1.2	124–188	2	1 + N	0
**UNH-174**	G12326	perfect	20	F* 48/1.5	146–187	4	1	0
**UNH-189**	G12341	perfect	12	R* 52/1.2	135–208	3	3	1
**UNH-190**	G12342	compound	21	R* 60/1.0	133–202	2	1	0
**UNH-197**	G12348	interrupted	23	R* 50/1.2	154–228	6	5	0
UNH-207	G12358	interrupted	6	R* 60/1.2	90–198	3	4	1
**UNH-211 **	G12362	perfect	19	R* 48/1.5	82–194	6	7	1
UNH-216	G12367	perfect	23	R* 52/1.2	126–212	2	4	0

				Average across loci		4.3	2.8	0.48
				Polymorphism *P *(0.95)		97%	77%	

**Table 3:** Test of balanced meiotic transmission of alleles from both parental species (On for *O. niloticus* and Sm for *S. melanotheron*) for each of the 8 hybrid F1 breeders (G1 (♀ On × ♂ Sm) and G′1 (♀ Sm × ♂ On)); Chi^2^ values and associate *P*-values are given; significant (i.e., biased transmission) tests (*a* = 0.05) when applying sequential Bonferroni correction (*n* = independent tests) are indicated in **bold**.

Families	BC-A		BC-B		BC-C		BC-D		F2-A				F2-B			
Breeders	♀ G1		♂ G1		♀ G**′**1		♂ G**′**1		♀ G**′**1		♂ G**′**1		♀ G1		♂ G**′**1	

# loci	28		28		24		23		6		8		9		9	

Allele origin	On	Sm	On	Sm	On	Sm	On	Sm	On	Sm	On	Sm	On	Sm	On	Sm
* n*_ exp_	698	698	698	698	597	597	573	573	150	150	198	198	225	225	225	225
* n*_ obs_	682	713	710	686	583	611	506	639	137	162	195	201	203	247	251	199
Chi^2^	0.689		0.413		0.657		15.449		2.09		0.091		4.302		6.009	
*P*	0.407		0.521		0.418		8.47**E** − 05		0.148		0.763		0.038		0.014	

**Table 4:** Comparisons of allelic distributions observed among the 4 backcross progeny; the number of loci implicated, the Fisher's test value and the associated *P*-values are given; significant heterogeneity (*a* = 0.05) is indicated in **bold** (see Table 1 for detail about hybrid types).

Comparisons	# Loci	Fisher's test	*P*
Global	25	65.51	0.069
Hybrids G1	26	44.91	0.747
Hybrids G′1	20	54.46	0.063
Hybrids ♀	24	49.08	0.429
Hybrids ♂	22	68.8	**0.010**
♂ G1/♀ G′1	23	42.82	0.606
♀ G1/♂ G′1	21	55.93	0.074

**Table 5:** Comparison of linkage associations expected from tilapia genetic maps and observed within the 7 studied families; linkage group (LG) is taken from the previously published genetic maps (A: [48]; B: [50]; C: [49]; D: [51]); the recombination rate (Rec) is given with the associated LOD score; significant linkages (LOD > 3) are represented in **bold**, while suggestive linkages (LOD > 2) are underlined; see Table 1 for detail about hybrid types.

Families					BC-A				BC-B				BC-C				BC-D				P-Sm			
Locus A	LG	Locus B	LG	References	♀ G1		♂ O. nil.		♀ O. nil.		♂ G1		♀ G′1		♂ O. nil.		♀ O. nil.		♂ G′1		♀ S. mel.		♂ S. mel.	
					Rec	LOD	Rec	LOD	Rec	LOD	Rec	LOD	Rec	LOD	Rec	LOD	Rec	LOD	Rec	LOD	Rec	LOD	Rec	LOD
UNH-008	17	UNH-103	17	A, D	0.42	0.28	0.42	0.28	0.46	0.07			0.35	0.9					0.42	0.28				
UNH-008	17	UNH-124	4		**0.10**	**7.74**	**0.08**	**8.73**	**0.20**	**3.97**	**0.09**	**7.95**	**0.06**	**9.58**					**0.22**	**3.61**				
UNH-008	17	UNH-146	4	B versus A, D	**0.08**	**9.00**	**0.02**	**12.92**			**0.00**	**15.05**	**0.00**	**14.45**					**0.04**	**11.4**				
UNH-102	16	UNH-125	16	A, C	0.36	0.86			0.28	2.18	0.26	2.61	0.3	1.79	0.32	1.44			0.36	0.86	**0.24**	**3.08**	**0.22**	**3.61**
UNH-102	16	UNH-138	16	A, C	0.38	0.63			0.28	2.18	0.38	0.63	0.36	0.86	0.4	0.44			0.41	0.3			0.46	0.07
UNH-106	3	UNH-207	6								**0.24**	**3.08**					0.44	0.16	0.42	0.28				
UNH-115	3	UNH-131	3	A, D	**0.1**	**7.99**					**0.10**	**7.99**	**0.04**	**10.56**	**0.23**	**3.04**			**0.02**	**12.05**				
UNH-115	3	UNH-135	3	A, D	**0.02**	**12.92**					**0.04**	**11.4**	**0.02**	**12.92**	**0.02**	**12.92**			**0.00**	**15.05**				
UNH-117		UNH-149	5		**0.02**	**12.34**					**0.00**	**15.5**							NT					
UNH-123	12	UNH-189	12	A, C, D	**0.12**	**7.08**	**0.24**	**3.08**	**0.12**	**6.84**	**0.18**	**4.6**	**0.2**	**4.19**					**0.12**	**7.08**				
UNH-124	4	UNH-146	4	C, D	**0.06**	**9.85**	**0.10**	**7.74**			**0.09**	**7.95**	**0.06**	**9.85**	**0.10**	**7.74**			**0.18**	**4.82**				
UNH-125	16	UNH-138	16	A, B, C, D	**0.02**	**12.92**	**0.08**	**9.00**	**0.00**	**15.05**	**0.12**	**7.08**	**0.06**	**10.12**	**0.08**	**9.00**	**0.00**	**13.85**	**0.04**	**10.27**			**0.24**	**3.08**
UNH-130	23	UNH-197	23	A, C	0.46	0.07									0.28	2.18			0.48	0.02				
UNH-130	23	UNH-216	23	A, C	0.33	1.31	**0.02**	**12.63**											0.44	0.16				
UNH-131	3	UNH-135	3	A, D	**0.08**	**9.00**	**0.04**	**11.4**			**0.06**	**10.12**	**0.02**	**12.05**	**0.21**	**3.58**			**0.02**	**12.05**			**0.04**	**11.12**
UNH-154	6	UNH-207	6	A versus D	**0.02**	**12.92**			**0.02**	**12.92**	**0.02**	**12.92**	**0.04**	**11.4**			**0.02**	**12.92**			**0.00**	**14.75**	**0.04**	**11.12**
UNH-197	23	UNH-216	23	A, D	**0.23**	**3.42**					0.20	4.19							0.08	9.00	0.13	**6.60**	**0.08**	**8.47**

## References

[1] Arnold ML (1997). *Natural Hybridization and Evolution*.

[2] Rieseberg LH, Willis JH (2007). Plant speciation. *Science*.

[3] Mallet J (2005). Hybridization as an invasion of the genome. *Trends in Ecology and Evolution*.

[4] Rieseberg LH, Raymond O, Rosenthal DM (2003). Major ecological transitions in wild sunflowers facilitated by hybridization. *Science*.

[5] Baack EJ, Rieseberg LH (2007). A genomic view of introgression and hybrid speciation. *Current Opinion in Genetics and Development*.

[6] Via S (2002). The ecological genetics of speciation. *American Naturalist*.

[7] Mallet J (2008). Hybridization, ecological races and the nature of species: empirical evidence for the ease of speciation. *Philosophical Transactions of the Royal Society B*.

[8] Arnold ML (2004). Natural hybridization and the evolution of domesticated, pest and disease organisms. *Molecular Ecology*.

[9] Bartley DM, Rana K, Immink AJ (2000). The use of inter-specific hybrids in aquaculture and fisheries. *Reviews in Fish Biology and Fisheries*.

[10] Seehausen O (2004). Hybridization and adaptive radiation. *Trends in Ecology and Evolution*.

[11] Seehausen O, Koetsier E, Schneider MV (2003). Nuclear markers reveal unexpected genetic variation and a Congolese-Nilotic origin of the Lake Victoria cichlid species flock. *Proceedings of the Royal Society B*.

[12] Joyce DA, Lunt DH, Genner MJ, Turner GF, Bills R, Seehausen O (2011). Repeated colonization and hybridization in Lake Malawi cichlids. *Current Biology*.

[13] Joyce DA, Lunt DH, Bills R (2005). An extant cichlid fish radiation emerged in an extinct Pleistocene lake. *Nature*.

[14] Schelly R, Salzburger W, Koblmüller S, Duftner N, Sturmbauer C (2006). Phylogenetic relationships of the lamprologine cichlid genus *Lepidiolamprologus* (Teleostei: Perciformes) based on mitochondrial and nuclear sequences, suggesting introgressive hybridization. *Molecular Phylogenetics and Evolution*.

[15] Rüber L, Meyer A, Sturmbauer C, Verheyen E (2001). Population structure in two sympatric species of the Lake Tanganyika cichlid tribe Eretmodini: evidence for introgression. *Molecular Ecology*.

[16] Salzburger W, Baric S, Sturmbauer C (2002). Speciation via introgressive hybridization in East African cichlids?. *Molecular Ecology*.

[17] Klett V, Meyer A (2002). What, if anything, is a Tilapia? Mitochondrial ND2 phylogeny of tilapiines and the evolution of parental care systems in the African cichlid fishes. *Molecular Biology and Evolution*.

[18] Schwarzer J, Misof B, Tautz D, Schliewen UK (2009). The root of the East African cichlid radiations. *BMC Evolutionary Biology*.

[19] Schliewen UK, Klee B (2004). Reticulate sympatric speciation in Cameroonian crater lake cichlids. *Frontiers in Zoology*.

[20] Rognon X, Guyomard R (2003). Large extent of mitochondrial DNA transfer from *Oreochromis aureus* to *O. niloticus* in West Africa. *Molecular Ecology*.

[21] Pouyaud L (1994). *Génétique des populations de tilapias d'intérêt aquacole en Afrique de l'Ouest. Relations phylogénétiques et structurations populationnelles*.

[22] Daget J, Moreau J (1981). Hybridation introgressive entre deux espèces de *Sarotherodon* (Pisces, Cichlidae) dans un lac de Madagascar. *Bulletin du Muséum National d'Histoire Naturelle*.

[23] Gregg RE, Howard JH, Shonhiwa F (1998). Introgressive hybridization of tilapias in Zimbabwe. *Journal of Fish Biology*.

[24] Wohlfarth GW (1994). The unexploited potential of tilapia hybrids in aquaculture. *Aquaculture & Fisheries Management*.

[25] Eknath AE, Hulata G (2009). Use and exchange of genetic resources of Nile tilapia (*Oreochromis niloticus*). *Reviews in Aquaculture*.

[26] Wohlfarth GW, Wedekind H (1991). The heredity of sex determination in tilapias. *Aquaculture*.

[27] Hulata G, Wohlfarth G, Rothbard S (1983). Progeny-testing selection of tilapia broodstocks producing all-male hybrid progenies—Preliminary results. *Aquaculture*.

[28] Mair GC, Scott AG, Penman DJ, Skibinski DOF, Beardmore JA (1991). Sex determination in the genus *Oreochromis*—2. Sex reversal, hybridisation, gynogenesis and triploidy in *O. aureus* Steindachner. *Theoretical and Applied Genetics*.

[29] Baroiller JF, D’Cotta H (2001). Environment and sex determination in farmed fish. *Comparative Biochemistry and Physiology C*.

[30] Cnaani A, Lee BY, Zilberman N (2008). Genetics of sex determination in tilapiine species. *Sexual Development*.

[31] Baroiller JF, D’Cotta H, Bezault E, Wessels S, Hoerstgen-Schwark G (2009). Tilapia sex determination: where temperature and genetics meet. *Comparative Biochemistry and Physiology*.

[32] Hulata G, Wohlfarth GW, Karplus I (1993). Evaluation of *Oreochromis niloticus×O. aureus* hybrid progeny of different geographical isolates, reared under varying management regimes. *Aquaculture*.

[33] Brummett RE, Halstrom ML, Dunham RA, Smitherman RO Development of biochemical dichotomous keys for identification of American populations of *Oreochromis aureus, O. mossambicus, O. niloticus, O. urolepis hornorum* and *red tilapia*.

[34] Stelkens R, Seehausen O (2009). Genetic distance between species predicts novel trait expression in their hybrids. *Evolution*.

[35] Chevassus B (1998). *Modification du phénotype sexuel et du mode de reproduction chez les poissons Salmonidés : inversion sexuelle hormonale, gynogenèse, hybridation interspécifique et polyploïdisation*.

[36] Chevassus B (1983). Hybridization in fish. *Aquaculture*.

[37] Chenuil A, Galtier N, Berrebi P (1999). A test of the hypothesis of an autopolyploid vs. allopolyploid origin for a tetraploid lineage: application to the genus Barbus (Cyprinidae). *Heredity*.

[38] Hubes CL, Hubes LC (1932). Apparent parthenogenesis in nature, in a form of fish of hybrid origin. *Science*.

[39] Schultz RJ (1969). Hybridization, unisexuality and polyploidy in the teleost *Poeciliopsis* (Poeciliidae). *American Naturalist*.

[40] Butlin RK (2005). Recombination and speciation. *Molecular Ecology*.

[41] Woram RA, McGowan C, Stout JA (2004). A genetic linkage map for Arctic char (*Salvelinus alpinus*): evidence for higher recombination rates and segregation distortion in hybrid versus pure strain mapping parents. *Genome*.

[42] Rogers SM, Isabel N, Bernatchez L (2007). Linkage maps of the dwarf and normal lake whitefish (*Coregonus clupeaformis*) species complex and their hybrids reveal the genetic architecture of population divergence. *Genetics*.

[43] Rogers SM, Campbell D, Baird SJE, Danzmann RG, Bernatchez L (2001). Combining the analyses of introgressive hybridisation and linkage mapping to investigate the genetic architecture of population divergence in the lake whitefish (*Coregonus clupeaformis*, Mitchill). *Genetica*.

[44] Baroiller JF, Bezault E, Bonnet S Production of 2 reciprocal intergeneric hybrids between Oreochromis niloticus and Sarotherodon melanotheron.

[45] Estoup A, Angers B, Carvalho GR (1998). Microsatellites and minisatellites for molecular ecology: theoretical and empirical considerations. *Advances in Molecular Ecology*.

[46] Jarne P, Lagoda PJL (1996). Microsatellites, from molecules to populations and back. *Trends in Ecology and Evolution*.

[47] Lee WJ, Kocher TD (1996). Microsatellite DNA markers for genetic mapping in *Oreochromis niloticus*. *Journal of Fish Biology*.

[48] Kocher TD, Lee WJ, Sobolewska H, Penman D, McAndrew B (1998). A genetic linkage map of a cichlid fish, the tilapia (*Oreochromis niloticus*). *Genetics*.

[49] Agresti JJ, Seki S, Cnaani A (2000). Breeding new strains of tilapia: development of an artificial center of origin and linkage map based on AFLP and microsatellite loci. *Aquaculture*.

[50] McConnell SKJ, Beynon C, Leamon J, Skibinski DOF (2000). Microsatellite marker based genetic linkage maps of *Oreochromis aureus* and *O. niloticus* (Cichlidae): extensive linkage group segment homologies revealed. *Animal Genetics*.

[51] Lee BY, Lee WJ, Streelman JT (2005). A second-generation genetic linkage map of tilapia (*Oreochromis spp.*). *Genetics*.

[52] Bezault E, Rognon X, Gharbi K, Baroiller JF, Chevassus B (2012). Microsatellites cross-species amplification across some African cichlids. *International Journal of Evolutionary Biology*.

[53] Marqueurs microsatellites: isolement à l'aide de sondes non-radioactives, caractérisation et mise au point. http://www.agroparistech.fr/svs/genere/microsat/microsat.htm.

[54] Estoup A, Gharbi K, SanCristobal M, Chevalet C, Haffray P, Guyomard R (1998). Parentage assignment using microsatellites in turbot (*Scophthalmus maximus*) and rainbow trout (*Oncorhynchus mykiss*) hatchery populations. *Canadian Journal of Fisheries and Aquatic Sciences*.

[55] Rice WR (1989). Anylizing tables of statistical tests. *Evolution*.

[56] Sokal R, Rohlf FJ (1995). *Biometry, the Principles and Practice of Statistics in Biological Research*.

[57] Danzmann RG LINKMFEX: Linkage analysis package for outcrossed mapping families with male or female exchange of the mapping parent. http://www.uoguelph.ca/~rdanzman/software/LINKMFEX.zip.

[58] http://www.poissons-afrique.ird.fr/faunafri/.

[59] Trewavas E (1983). *Tilapiine Fishes of the Genera Sarotherodon, Oreochromis and Danakilia*.

[60] Philippart J-C, Ruwet JC, Pullin RSV, Lowe-McConnell RH (1982). Ecology and distribution of tilapias. *The Biology and Culture of Tilapias*.

[61] Lemarié G, Baroiller JF, Clota F, Lazard J, Dosdat A (2004). A simple test to estimate the salinity resistance of fish with specific application to *O. niloticus* and *S. melanotheron*. *Aquaculture*.

[62] Hulata G, Cnaani A, Slossman T, Gall GAE (2004). Fertility problems in the second generation of a four-species tilapia cross. *Israeli Journal of Aquaculture*.

[63] Dobzhansky T (1936). Studies on hybrid sterility. II. Localization of sterility factors in *Drosophila pseudoobscura* hybrids. *Genetics*.

[64] Coyne JA, Orr AH (2004). *Speciation*.

[65] Maan ME, Seehausen O, Söderberg L (2004). Intraspecific sexual selection on a speciation trait, male coloration, in the Lake Victoria cichlid Pundamilia nyererei. *Proceedings of the Royal Society B*.

[66] Keenleyside MHA, Keenleyside M (1991). Parental care. *Cichlid Fishes: Behaviour, Ecology and Evolution*.

[67] Barlow GW, Keenleyside M (1991). Mating systems among cichlid fishes. *Cichlid Fishes: Behaviour, Ecology and Evolution*.

[68] Stelkens RB, Schmid C, Selz O, Seehausen O (2009). Phenotypic novelty in experimental hybrids is predicted by the genetic distance between species of cichlid fish. *BMC Evolutionary Biology*.

[69] Stelkens RB, Young KA, Seehausen O (2010). The accumulation of reproductive incompatibilities in African cichlid fish. *Evolution*.

[70] Seehausen O, Van Alphen JJM, Witte F (1997). Cichlid fish diversity threatened by eutrophication that curbs sexual selection. *Science*.

