# Supplementary material for: Analysis of the Meiotic Segregation in Intergeneric Hybrids of Tilapias
Source: Int J Evol Biol. 2012 Jun 18;2012:817562. doi: 10.1155/2012/817562 (PMC3385623; doi:10.1155/2012/817562)
Supplement: Supplementary file 1 — The supplementary material provides the details about Informative marker segregations and segregation distortions observed in the different families studied (Table S1); the adjustment to Mendelian expectations of the genotype distributions (Table S2); and the comparisons of the recombination rate between the different genetic types (Table S3). [file 817562.f1.pdf]

TABLE S1: Informative marker segregations and segregation distortions observed in the different families studied: back-cross, F2 hybrid & pure-species; Chi^2 (1df) values and associate P-values are given only for the test reaching a non-corrected threshold a $\leq$ 0.05, the non-informative segregations (*i.e.* fixed marker or homozygote in a given family) are noted with "-", while non-tested ("n.t.") segregations in F2 families are indicated in dark-grey; the origin of alleles loss is indicated by On for *O. niloticus* and Sm for *S. melanotheron*. Tests are considered significant (*) only if their P-value is smaller than the corrected threshold after Sequential Bonferroni procedure performed i) over segregations of each breeder (represented in **bold**) and ii) over all studied segregations for each locus (represented as underlined)

Families		BC-A		BC-B		BC-C		BC-D		F2-A		F2-B		P-Sm	
Loci	LG	♀ G1	♂ On	♀ On	♂ G1	♀ G'1	♂ On	♀ On	♂ G'1	♀ G'1	♂ G'1	♀ G1	♂ G'1	♀ Sm	♂ Sm
**UNH-008**	17			5.21 *			-	-	**On 10.02** **	-	-			-	-
**UNH-102**	16		-					-		On 3.92 *		-	-		
UNH-103	17				-		-			n.t.	n.t.	n.t.	n.t.	-	-
UNH-106	3	-		-		-	-			n.t.	n.t.	n.t.	n.t.	-	-
UNH-115	3		-	-				-		n.t.	n.t.	n.t.	n.t.	-	-
UNH-117			-	-		-	-	-	-	n.t.	n.t.	n.t.	n.t.	-	-
UNH-123	12									n.t.	n.t.	n.t.	n.t.	-	
UNH-124	4			4.33 *				-		n.t.	n.t.	n.t.	n.t.	-	-
UNH-125	16				Sm 5.21 *					n.t.	n.t.	n.t.	n.t.		
**UNH-129**	8		-						-						
UNH-130	23			-	-	-				n.t.	n.t.	n.t.	n.t.	-	-
**UNH-131**	3			-				-		-				-	
UNH-132	9			-			-	-	On 4.67 *	n.t.	n.t.	n.t.	n.t.	-	-
UNH-135	3			-				-		n.t.	n.t.	n.t.	n.t.	8.23 **	
UNH-138	16									n.t.	n.t.	n.t.	n.t.	-	
**UNH-142**								-		-					-
**UNH-146**	4			-				-	On 6.63 **			-		-	-
**UNH-149**	5		-	-		Sm 3.97 *				-				-	-
**UNH-154**	6						-		-	-			-		
UNH-159	2							-		n.t.	n.t.	n.t.	n.t.	-	-
**UNH-162**	4		-			On 6.63 **					On 5.12 *		Sm 5.12 *	-	
UNH-169	5		-	-			-			n.t.	n.t.	n.t.	n.t.	-	
UNH-173	13	-	-	-		-	-	-		n.t.	n.t.	n.t.	n.t.		-
**UNH-174**	20		-	-	On 6.63 **			-		-				-	-
**UNH-189**	12						-	-		-	-		Sm 6.48 *		-
**UNH-190**	21			-	On 5.21 *			-		-				-	-
**UNH-197**	23		-			-		-	On 6.63 **			**On 8** **			
UNH-207	6		-				-			n.t.	n.t.	n.t.	n.t.		
**UNH-211**	19		-		On 8.23 **										
UNH-216	23			-			-	-	**On 10.02** **	n.t.	n.t.	n.t.	n.t.		
**# informative Segregations**		28	18	16	28	25	19	13	27	6	8	9	9	12	14

TABLE S2: Adjustment to Mendelian expectations of the genotype distributions observed in the two studied hybrid F2 families; both Chi^2 and Fisher's test values and associate p-values are given, significant distortions (a = 0.05) are indicated in **bold**

**Families**	**F2-A**		**F2-B**		**Total Families / Locus**	
**Locus**	**Chi2**	**p**	**Chi2**	**p**	**Fisher's test**	**p**
UNH-008	1.82	0.403	6	0.112	6.2	0.185
UNH-102	4.72	0.193	2	0.368	5.29	0.259
UNH-129	2.64	0.451	0.36	0.835	1.95	0.744
UNH-131	1.55	0.46	1.68	0.641	2.44	0.655
UNH-142	3.44	0.179	0.24	0.971	3.5	0.478
UNH-146	7.57	0.056	1.32	0.517	7.09	0.131
UNH-149	1.32	0.517	3.92	0.27	3.94	0.415
UNH-154	0.72	0.396	0.08	0.777	2.36	0.671
UNH-162	5.84	0.12	7.44	0.059	**9.9**	**0.042**
UNH-174	2.16	0.34	0.24	0.887	2.4	0.663
UNH-189	0.55	0.759	6.64	0.084	5.5	0.24
UNH-190	3.88	0.144	0.24	0.887	4.12	0.39
UNH-197	2.8	0.423	**12.88**	**0.005**	**12.35**	**0.015**
UNH-211	2.16	0.54	2.16	0.54	2.47	0.651
**Total Loci / Family**						
**Fisher's test**	34.42	0.187	35.09	0.167	63.09	0.24

TABLE S3: Comparisons of the recombination rate between the different genetic types, a) pure species (*O. niloticus* & *S. melanotheron*) and b) F1 hybrids (G1 & G'1), for both sexes; linkage group (LG) are taken from the previously published genetic maps (A: [48]; B: [50]; C: [49]; D: [51]); Chi^2 values and associate uncorrected *P*-values are given, significant tests (a = 0.05) when applying sequential Bonferroni correction (based on the number of loci tested for each comparison model) are indicated in **bold**

a) Pure species

Locus A	LG	Locus B	LG	Comparison	*O.nil.* ♀			*O.nil.* ♂			*O.nil.* ♂ & ♀			*S.mel.* ♂ & ♀			*O.nil.* & *S.mel.*		
				Reference	d.f.	Chi2	*P*	d.f.	Chi2	*P*	d.f.	Chi2	*P*	d.f.	Chi2	*P*	d.f.	Chi2	*P*
UNH 008	17	UNH 103	17	A,D							1	0.16	0.69				1	0.16	0.69
UNH 008	17	UNH 124	4								1	2.61	0.11				1	2.61	0.11
UNH 008	17	UNH 146	4	*B vs A,D*															
UNH 102	*16*	UNH 125	16	A, C							1	0.19	0.66	1	0.06	0.81	3	1.51	0.68
UNH 102	*16*	UNH 138	16	A, C							1	1.60	0.21				2	3.57	0.17
UNH 106	3	UNH 207	6																
UNH 115	3	UNH 131	3	A, D															
UNH 115	3	UNH 135	3	A, D															
UNH 117		UNH 149	5																
UNH 123	12	UNH 189	12	A, C, D							1	2.30	0.13				1	2.30	0.13
UNH 124	4	UNH 146	4	C, D				1	0.00	1.00							1	0.00	1.00
UNH 125	16	UNH 138	16	A, B, C, D	1	0.00	1.00	1	0.00	1.00	3	8.01	0.05				**4**	**25.36**	**0.00**
UNH 130	*23*	UNH 197	23	A, C															
UNH 130	*23*	UNH 216	23	A, C															
UNH 131	3	UNH 135	3	A, D				**1**	**6.67**	**0.01**							**2**	**10.92**	**0.00**
UNH 154	*6*	UNH 207	6	*A vs D*	1	0.00	1.00							1	2.04	0.15	3	2.06	0.56
UNH 197	23	UNH 216	23	A, D										1	0.45	0.50	1	0.45	0.50

b) F1 Hybrids

Locus A	LG	Locus B	LG	Comparison	G1 & G'1			G1 ♂ & ♀			G'1 ♂ & ♀			Hybrids ♀			Hybrids ♂		
				Reference	d.f.	Chi2	*P*	d.f.	Chi2	*P*	d.f.	Chi2	*P*	d.f.	Chi2	*P*	d.f.	Chi2	*P*
UNH 008	17	UNH 103	17	A,D	2	0.59	0.74				1	0.45	0.50	1	0.45	0.50			
UNH 008	17	UNH 124	4		3	6.90	0.08	1	0.06	0.81	1	4.96	0.03	1	0.50	0.48	1	3.22	0.07
UNH 008	17	UNH 146	4	*B vs A,D*	7	19.59	0.01	2	8.96	0.01	4	10.60	0.03	3	8.42	0.04	3	11.04	0.01
UNH 102	*16*	UNH 125	16	A, C	3	1.65	0.65	1	1.17	0.28	1	0.41	0.52	1	0.41	0.52	1	1.17	0.28
UNH 102	*16*	UNH 138	16	A, C	3	0.29	0.96	1	0.00	1.00	1	0.28	0.60	1	0.04	0.84	1	0.11	0.74
UNH 106	3	UNH 207	6		1	3.66	0.06										1	3.66	0.06
UNH 115	3	UNH 131	3	A, D	3	3.76	0.29	1	0.00	1.00	1	0.34	0.56	1	1.19	0.28	1	2.59	0.11
UNH 115	3	UNH 135	3	A, D	3	2.04	0.56	1	0.34	0.56	1	1.01	0.31	1	0.00	1.00	1	2.04	0.15
UNH 117		UNH 149	5		1	1.05	0.31	1	1.05	0.31									
UNH 123	12	UNH 189	12	A, C, D	3	2.01	0.57	1	0.78	0.38	1	1.19	0.28	1	1.19	0.28	1	0.78	0.38
UNH 124	4	UNH 146	4	C, D	3	5.37	0.15	1	0.23	0.63	1	3.28	0.07	1	0.00	1.00	1	1.77	0.18
UNH 125	16	UNH 138	16	A, B, C, D	3	4.74	0.19	1	3.84	0.05	1	0.13	0.72	1	1.04	0.31	1	1.84	0.17
UNH 130	*23*	UNH 197	23	A, C	1	0.04	0.84												
UNH 130	*23*	UNH 216	23	A, C	1	1.35	0.25												
UNH 131	3	UNH 135	3	A, D	3	2.83	0.42	1	0.15	0.70	1	0.00	1.00	1	1.71	0.19	1	0.92	0.34
UNH 154	*6*	UNH 207	6	*A vs D*	2	0.51	0.77	1	0.00	1.00				1	0.34	0.56			
UNH 197	23	UNH 216	23	A, D	2	4.26	0.12	1	0.09	0.76							1	2.99	0.08
